# Unusual Types of Plasma Cell Dyscrasias Associated With HIV: Polyneuropathy, Organomegaly, Endocrinopathy, M-protein, and Skin Changes (POEMS) Syndrome

**DOI:** 10.7759/cureus.62820

**Published:** 2024-06-21

**Authors:** Mohamed Reda Belkhribchia, Zaineb Baroudi, Abdelaziz Ajrinija, Itab Ikane

**Affiliations:** 1 Department of Neurology, Hassan II Regional Hospital, Dakhla, MAR; 2 Department of Radiology, Hassan II Regional Hospital, Dakhla, MAR; 3 Department of Rheumatology, Hassan II Regional Hospital, Dakhla, MAR; 4 Department of General Practice, Hassan II Regional Hospital, Dakhla, MAR

**Keywords:** hiv aids, poems syndrome, igm lambda paraprotein, lenalidomide, dexamethasone, nerve biopsy, autologous hematopoietic stem cell transplantation

## Abstract

Polyneuropathy, organomegaly, endocrinopathy, M-protein, and skin changes (POEMS) syndrome is a multisystem paraneoplastic disorder due to an underlying plasma cell neoplasm, and its occurrence among HIV patients is extremely rare. The diagnosis of POEMS syndrome can be challenging in this context, particularly if its disabling polyneuropathy is misdiagnosed as neuropathy related to HIV. Herein, we report the case of a female patient with treated HIV who later developed POEMS syndrome. After a misdiagnosis of chronic inflammatory demyelinating polyneuropathy related to HIV and unsuccessful corticosteroids and cyclophosphamide therapies, the correct diagnosis of POEMS syndrome was made. The patient achieved significant hematological and neurological improvement after six cycles of lenalidomide. Autologous stem cell transplantation was then scheduled to prevent eventual relapses.

## Introduction

Polyneuropathy, organomegaly, endocrinopathy, M-protein, and skin changes (POEMS) syndrome is a rare and disabling multisystem paraneoplastic syndrome due to an underlying plasma cell neoplasm. A wide range of malignancies have been reported in people living with HIV/AIDS (PLWHA). Nonetheless, the occurrence of POEMS syndrome in PLWHA is exceedingly rare and was reported only once in the literature [1]. 

Herein, we report the case of a young female patient with a history of treated HIV who later developed POEMS syndrome. The diagnosis of this association can be challenging, particularly if the disabling neuropathy of POEMS syndrome is misdiagnosed as an HIV-related neurological complication. Some red flags should alert the clinician to this diagnosis, which is of utmost importance due to the treatability of POEMS syndrome.

## Case presentation

In February 2023, a 36-year-old female patient-the mother of four children-began complaining about intense neuropathic pain in the lower limbs (LLs) and then in the upper limbs (ULs). Her medical history was relevant for HIV infection treated with highly active antiretroviral therapy (HAART, dolutegravir/lamivudine/tenofovir) for a year. A secondary amenorrhea for six months was also reported by the patient. The neurologic symptoms had a subacute course, beginning with calf cramps, tingling, and burning pain in the feet, followed by a weakness in the same location. Two weeks later, similar symptoms occurred in the hands. These symptoms continued worsening relentlessly during the following weeks, in addition to weight loss. In late April 2023, the patient presented for the first time to our department, and the physical examination revealed a severe distal motor weakness affecting bilaterally both feet and hands, which left the patient wheelchair dependent. The Medical Research Council (MRC) score evaluated the weakness at 1/5 at the distal extremities of LL and 2/5 at the distal muscles of UL. Sensory loss of all modalities was also evident in the distal parts of UL and LL. The deep tendon reflexes were preserved except for the Achilles reflexes, which were abolished. 

Nerve conduction studies (NCS) revealed no motor or sensory responses in LL. A mixed pattern of demyelinating and axonal, sensory and motor neuropathy affecting the median nerves in UL was recorded. The needle electromyography showed intense acute denervation, especially in the distal muscles of the four limbs. Cerebrospinal fluid (CSF) revealed 15 white cells/mm3 with an increased concentration of protein at 0.77 g/l (normal range <0.45 g/l). The CSF examination for opportunistic infections, such as *Mycobacterium tuberculosis* and *Cryptococcus neoformans* via polymerase chain reaction (PCR), was unrevealing. In serum, the viral load of HIV and CD4 cell counts were, respectively, 61 copies/ml and 478 cells/mm^3^. 

**Figure 1:**
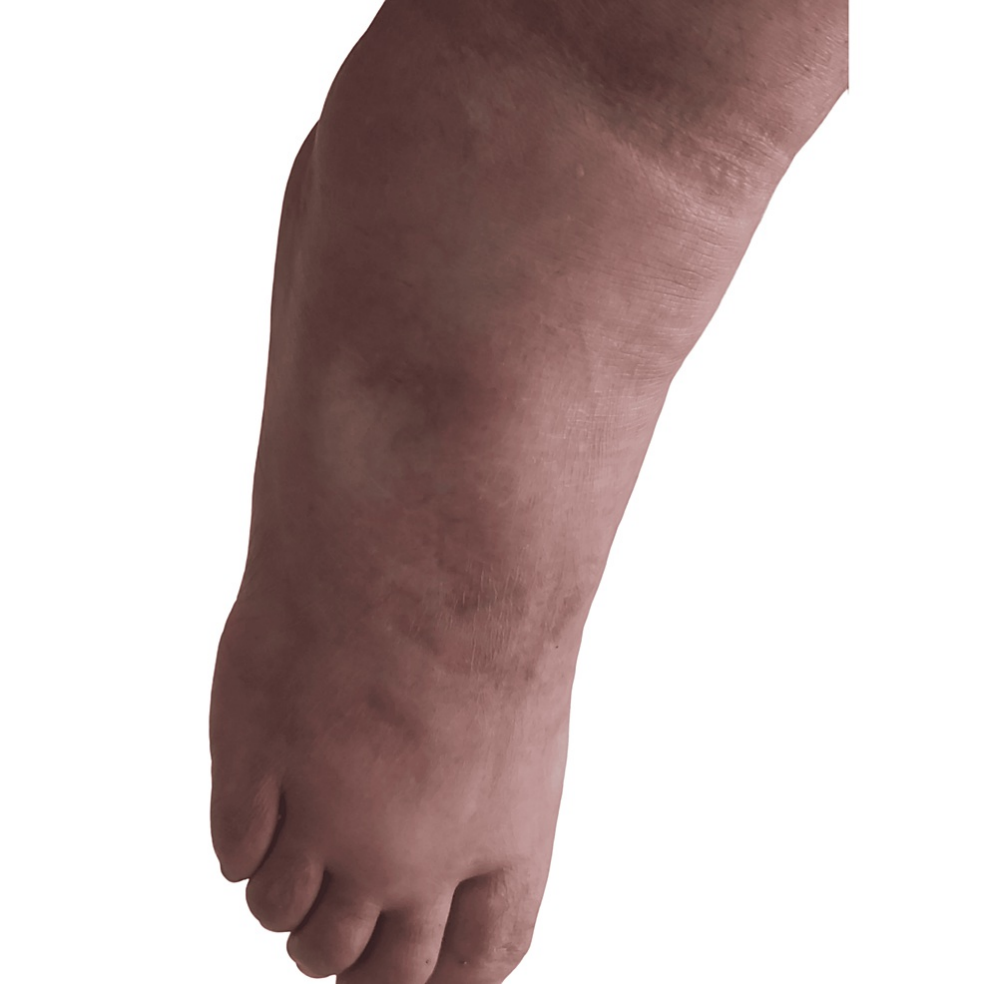
Edema of the foot.

**Figure 2:**
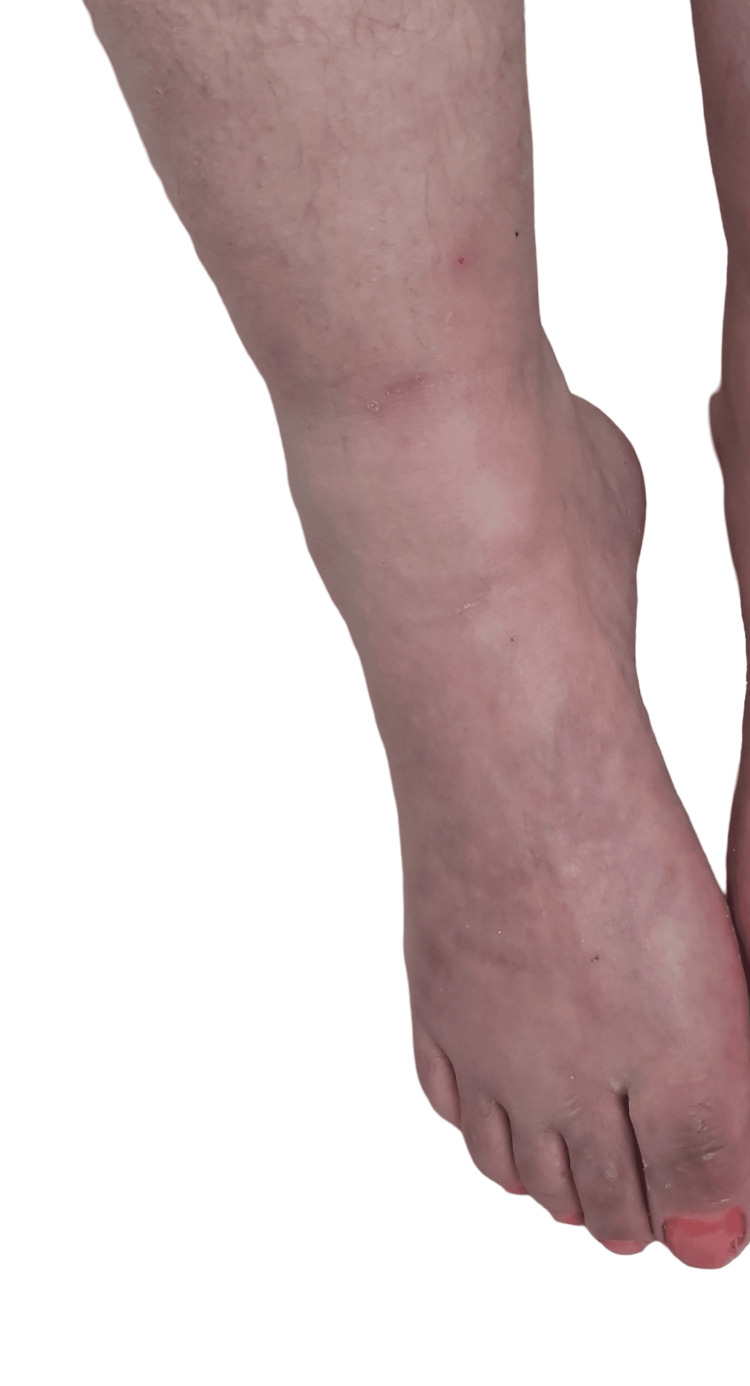
Acrocyanosis of the foot.

**Figure 3:**
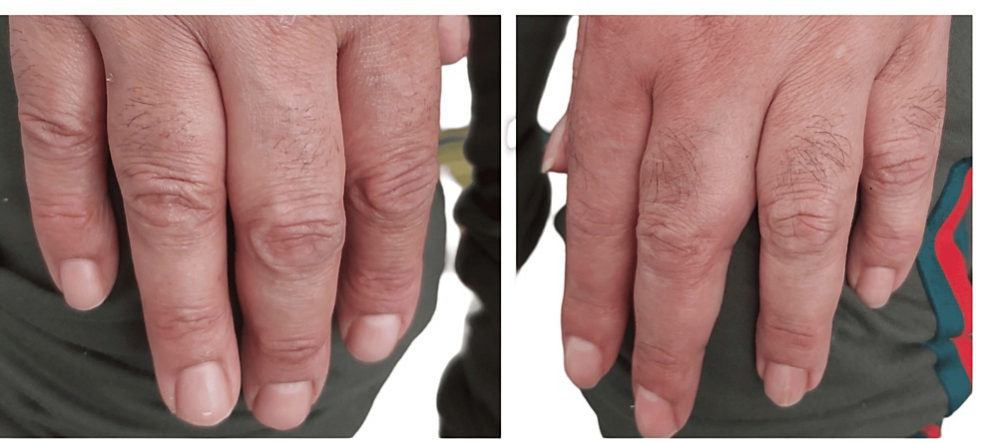
Left hand: hypertrichosis (normal pilosity in the right hand).

After this initial evaluation, the diagnosis of a possible distal variant of chronic inflammatory demyelinating polyneuropathy (CIDP) triggered by HIV infection was made. Attempting to obtain an improvement in polyneuropathy, we started high-dose pulse methylprednisolone with a scheme of one gram daily for five consecutive days. Then, we switched to oral prednisolone at 1 mg/kg/day for six weeks, followed by a slow tapering until a daily maintenance dose of 10 mg. In June 2023, six weeks after the onset of this therapeutic scheme, the patient experienced some improvement in dysesthesia. Nevertheless, no improvement in sensory and motor deficits was noted. Subsequently, two grams of parenteral cyclophosphamide were administered in July and August 2023, but without significant neurological improvement. Moreover, the patient developed intermittent edema of the feet, which worsened at night. We noticed the appearance of a permanent erythematous and violaceous discoloration of the hands, the feet, and the face, indicating an acrocyanosis. Hypertrichosis over the dorsal surface of the left hand also developed. A mild digital clubbing was also present (Figures 1-3). 

Notably, the patient also experienced during this period three transient ischemic attacks (TIAs) manifesting as episodes of transient right monocular blindness, which lasted approximately from one to two hours per episode. To prevent further vascular attacks, we added aspirin 100 mg/day. Nevertheless, aspirin was switched to clopidogrel 75 mg/day due to the persistence of TIA under aspirin. 

**Figure 4:**
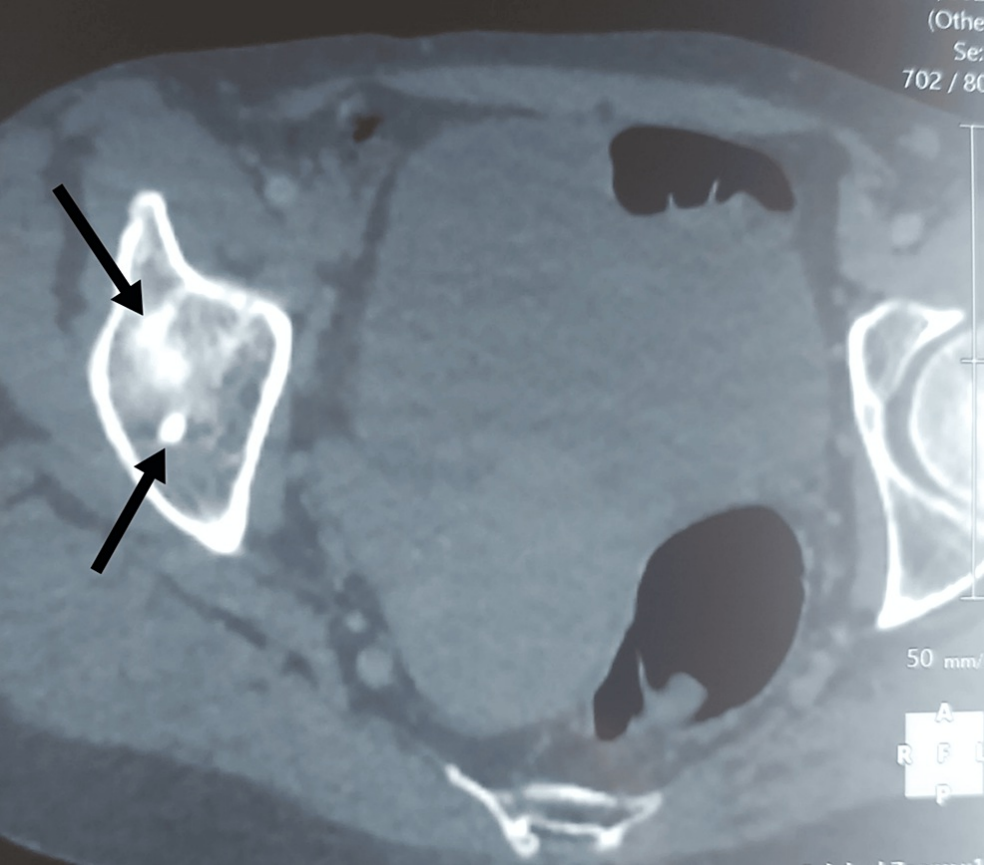
CT scan of the pelvis shows densely sclerotic bone lesions in the right acetabulum (black arrows). CT: computed tomography.

The lack of response to polyneuropathy despite high doses of corticosteroids and additional cyclophosphamide, in addition to the occurrence of new systemic symptoms, led us to reconsider the diagnosis of CIDP related to HIV. Thus, we performed more investigations to identify the actual underlying disease responsible for the whole clinical picture. Cardiologic (electrocardiogram and echocardiogram), renal, and hepatic tests were unremarkable, and therefore, excluding a common etiology of LL edema. A hormonal workup for secondary amenorrhea revealed a low estradiol level at 20.3 pg/ml (normal range: 30-400 pg/ml for premenopausal women) with normal follicle-stimulating hormone (FSH), indicating hypogonadotropic hypogonadism. Thyroxine, thyroid stimulating hormone (TSH), cortisol, and prolactin were within normal range. Magnetic resonance imagery (MRI) of the brain was unremarkable. Serum electrophoresis revealed low albumin at 30.6g/l (normal range: 37-49g/l), whereas gamma-globulins and beta1-globulins were elevated, respectively, at 26.6g/l (normal range: 7.4-14g/l) and 8.47 g/l (normal range: 3.1-5.3 g/l). Two monoclonal paraproteins, IgG lambda and IgM lambda, were revealed in serum immunofixation. Serum-free light chains (FLCs) showed both increased lambda and kappa chains, respectively, at 97.84 mg/l (normal: 5.71 to 26.30) and 106.29 mg/l (normal: 3.30 to 19.40). The kappa/lambda ratio was normal at 1,09 (normal: 0,26 to 1,65). The Bence-Jones protein urine test was negative. The bone marrow biopsy found no evidence of multiple myeloma. Radiography and computed tomography (CT) of the chest, abdomen, and pelvis showed several sclerotic bone lesions located in the sacrum, pelvis, and upper extremities of the femurs (Figure 4). 

Moderate ascites was also revealed, whereas no organomegaly was observed. The full blood count, hepatitis virus panel, and antithyroid autoantibodies were unremarkable. Vitamins B12, B9, auto-immunity workup (antinuclear and anti-DNA antibodies), in addition to viscosity evaluation (antiphospholipid antibodies, proteins C and S), were unremarkable. Serum vascular endothelial growth factor (VEGF) was increased to 544 pg/ml (normal range <500 pg/ml). The ophthalmologic examination was unremarkable. Sural nerve biopsy (analyzed by electron microscopy) unfolded a massive and diffuse loss of myelinated fibers; the scarce remaining myelinated fibers were transformed into ovoids, indicating acute axonal degeneration. Neovascularization into the vessels of the epineurium was observed (the vessel walls were non-necrotic). An inflammatory T cell infiltrate was noted, particularly in the epineurium (and slightly in the endoneurium). Hypertrophy of the endothelial cells in the capillaries of the endoneurium with the opening of the tight junctions between endothelial cells was also evident. 

This constellation of clinical, laboratory, and radiologic findings confirmed the diagnosis of POEMS syndrome in our patient. Indeed, the diagnostic criteria of POEMS syndrome were fulfilled with two mandatory major criteria (polyneuropathy, monoclonal plasma cell-proliferative disorder, which was lambda light-chain restricted); two major criteria (sclerotic bone lesions and VEGF elevation); and three minor criteria (extravascular volume overload as peripheral edema and ascites, endocrinopathy as hypogonadotropic hypogonadism and skin changes as acrocyanosis and hypertrichosis) (Table 1). 

**Table 1:** Diagnostic criteria for POEMS syndrome. Reference: [2]. VEGF: vascular endothelial growth factor, POEMS: Polyneuropathy, organomegaly, endocrinopathy, M-protein, and skin changes.

Criteria	
Mandatory major criteria—both required	Polyneuropathy (typically demyelinating)
	Monoclonal plasma cell disorder (typically lambda light-chain restricted)
Other major criteria—one required	Castleman disease
	Sclerotic bone lesions
	Elevated VEGF
Minor criteria—one required	Organomegaly
	Splenomegaly, hepatomegaly, lymphadenopathy
	Extravascular volume overload
	Peripheral edema, ascites, pleural effusions
	Endocrinopathy
	Adrenal, pituitary, gonadal, parathyroid
	Thyroid and pancreatic (nondiagnostic)
	Skin changes
	Hyperpigmentation, hypertrichosis, glomeruloid hemangiomata, plethora, acrocyanosis, flushing, white nails
	Papilledema
	Thrombocytosis or polycythemia

The patient had other manifestations of POEMS syndrome (non-diagnostic criteria), such as recurrent ischemic strokes, digital clubbing, and weight loss. After hematology advice, and despite the episodes of TIA, lenalidomide was initiated (in September 2023) in association with prophylactic rivaroxaban 15 mg/day and clopidogrel 75 mg/day. Lenalidomide (LD) was given at 25 mg daily from day one to day twenty-one each cycle of 28 days. Due to the previous high doses of corticosteroids, dexamethasone (Dex) was used only at 10 mg weekly (instead of 40 mg/week). 

**Figure 5:**
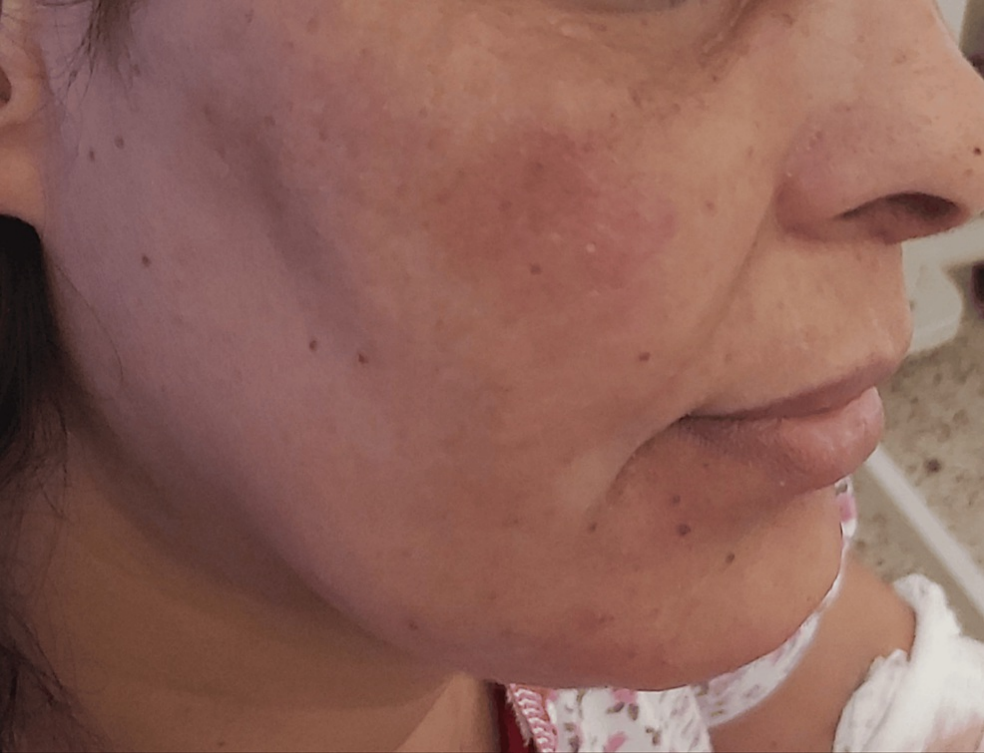
Before the initiation of lenalidomide: acrocyanosis and plethora of the face.

**Figure 6:**
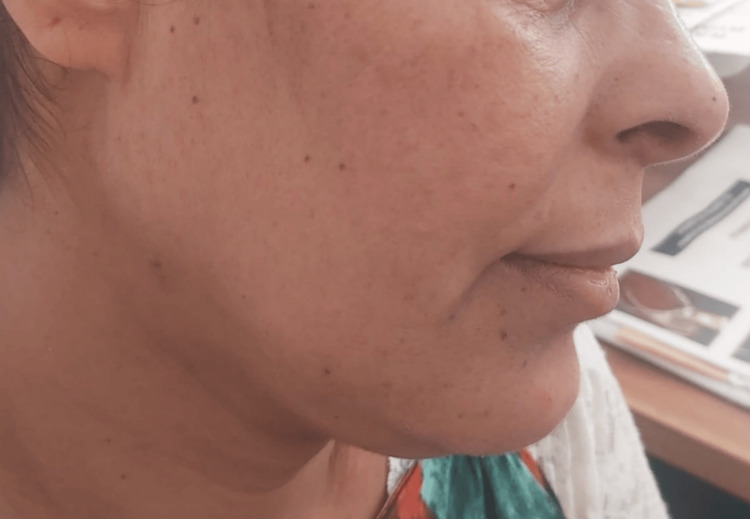
After six cycles of lenalidomide: the disappearance of the acrocyanosis and plethora of the face.

After six cycles of LD-Dex, a significant neurological response was obtained with the improvement of the distal motor weakness of the LL from 1/5 to 4/5 and that of the hands from 2/5 to 4/5, according to the MRC score. Sensory deficit also improved. The improvement of the polyneuropathy allowed the patient to walk using a cane. The clinical response was also obvious regarding the other systemic manifestations. Indeed, we noticed that acrocyanosis, hypertrichosis, and edema of the lower limbs all had disappeared (Figures 5, 6). 

Moreover, since the initiation of the LD-Dex protocol, no longer TIA episode was recorded. Hypogonadism, however, did not respond completely (although the patient began experiencing some irregular and short menstruations). Hence, hormone replacement therapy was indicated. After six cycles of LD-Dex, serum immunofixation turned negative. Due to the mild increase in initial VEGF, its dosage was not redone.

The significant clinical and hematological response to the LD-Dex protocol allowed us to envision an autologous stem cell transplantation (ASCT) as a consolidation modality to prevent eventual relapses. The rehabilitation program was continued.

## Discussion

Our case is well illustrated by the famous idiom ‘‘the tree often hides the forest’’. In metaphorical terms, HIV “the tree” had hidden POEMS syndrome “the forest”. Our patient developed a subacute severe sensorimotor polyneuropathy, which was initially misdiagnosed as a possible distal variant of CIDP related to HIV. The first red flag against this assumption was the unresponsiveness of the polyneuropathy to high doses of corticosteroids and cyclophosphamide, which is very unusual in CIDP associated with HIV. Indeed, the existing literature reveals that treated HIV-infected patients with CIDP have a short duration of disease, have a benign course, and are highly corticosteroid-responsive [3]. The subsequent occurrence of systemic manifestations such as skin changes (acrocyanosis and hypertrichosis), peripheral edema, and recurrent ischemic strokes were additive red flags and encouraged us to work up for POEMS syndrome. 

Plasma cell dyscrasias are more common among PLWHA and have a varied clinicopathological spectrum. They tend to occur at younger ages and to be more aggressive among this population [4]. Nevertheless, to our best knowledge, the occurrence of POEMS syndrome in a patient with a history of treated HIV infection was reported only once [1]. Castleman disease (CD), also known as angiofollicular or giant lymph node hyperplasia, is a rare lymphoproliferative disorder, and its association with HIV and the human herpes virus (HHV)-8 is well known. Paradoxically, and despite the presence of CD in roughly 11 to 30% of POEMS patients [2], the association between POEMS syndrome and HIV is extremely rare. 

The CSF in POEMS syndrome reveals albuminocytological dissociation (ACD) and is typically acellular with raised protein concentration. Although the patient had a mild pleocytosis, the CSF responds to the definition of ACD in our case. Indeed, ACD is defined as an increased protein level (>0.45 g/L) in the absence of elevated white cell count (<50 cells/μL). Due to the exclusion of the main opportunistic infections via PCR in our case, this mild pleocytosis corresponds to aseptic meningitis, which is common and usually asymptomatic among HIV patients [5]. 

Although a nerve biopsy study is not mandatory in POEMS syndrome, we performed a sural nerve biopsy in our case. The pathological examination was beneficial in this context of immunodeficiency. Indeed, it ruled out some differentials, such as lymphomatosis, vasculitis, and amyloidosis. Moreover, it demonstrated the characteristic neuropathological findings in POEMS syndrome as described in previous reports [6-8]. Monoclonal plasma cell disorder represents the second mandatory diagnostic criteria of POEMS syndrome, which are typically IgA or IgG lambda-restricted. Our patient developed two monoclonal paraproteins: IgG lambda and IgM lambda. Notably, the nature of the monoclonal plasma cell disorder in our case is very unusual in POEMS syndrome. Firstly, IgG lambda and IgM lambda do not represent a true biclonal gammopathy but rather a unique clone that produced two monoclonal proteins with different heavy chains but identical light chains [9]. On the other hand, the heavy chain mu (IgM) is extremely rare as a component of the monoclonal gammopathy in POEMS syndrome. 

Regarding the other major diagnostic criteria, our patient had both elevated VEGF and sclerotic bone lesions. VEGF, a potent proinflammatory angiogenic cytokine, is a key biomarker in diagnosis and disease monitoring of the disease. It is highly raised in pre-therapeutic cases of POEMS syndrome [10]. Our case had a mildly increased level of VEGF, which is attributed to the effect of high doses of corticosteroids and pulses of cyclophosphamide administered before the dosage. Indeed, falsely negative (suppressed) VEGF levels may be found in patients recently treated with steroids for incorrectly diagnosed CIDP [11]. 

As mentioned above, our patient had three out of the six minor diagnostic criteria for POEMS syndrome (skin changes, extravascular volume overload, and endocrinopathy). Excepting endocrinopathy (hypogonadism with secondary amenorrhea), skin changes, peripheral edema, and TIA occurred approximately two months from the onset of the polyneuropathy. Indeed, POEMS syndrome often manifests initially as an isolated polyneuropathy or is associated with minimal systemic manifestations. Thus, the full-blown picture of POEMS syndrome may only be evident after several weeks or months from the beginning of the polyneuropathy. Our patient had other manifestations suggestive of POEMS syndrome, though non-diagnostic criteria, such as ischemic strokes, mild digital clubbing, and weight loss. These manifestations can also include hyperhidrosis, pulmonary hypertension/restrictive lung disease, diarrhea, and low vitamin B12 values. 

Ischemic stroke is a well-known association of POEMS syndrome. The estimated five-year ischemic stroke risk was 13.4% in a cohort of 90 patients. Risk factors include thrombocytosis and the presence of bone marrow plasmacytosis [12]. Our patient experienced three episodes of TIA before the initiation of LD, and notably, no ischemic episode was recorded since the administration of LD. This supports that the best approach for mitigating the vascular risk in POEMS syndrome is the effective treatment of the underlying plasma cell clone. 

LD, an immunomodulatory drug derivative of thalidomide, is an interesting option in POEMS syndrome due to its lack of neurologic toxicity, its effectiveness against plasma cell proliferations and its anti-VEGF effect. To minimize the prothrombotic effect of LD, rivaroxaban and clopidogrel were maintained during the period of the administration of this molecule. LD resulted in rapid neurological and systemic improvement in our patient, similar to previous reports [13,14]. As expected, this significant clinical improvement was observed in parallel to the hematological response following the LD-Dex protocol. 

The favorable course in our case is also due to the relatively short lapse of time separating the onset of polyneuropathy and the beginning of LD, which did not exceed six months. Indeed, the diagnosis of POEMS syndrome is typically delayed by 12-16 months, by which time patients can be severely disabled and bed- or wheelchair-bound with established neuropathy [11]. Due to the disseminated form of the disease with several bone lesions, ASCT was indicated. This therapeutic procedure aims to lower the risk of relapses after the cessation of LD. 

## Conclusions

Our case highlights that POEMS syndrome can coexist with HIV. This association can be challenging to diagnose, particularly if the polyneuropathy of POEMS syndrome is misdiagnosed as neuropathy related to HIV. The red flags alerting to the possibility of a coexisting POEMS syndrome with HIV are the subacute and severe motor deficit with striking distal localization and systemic manifestations such as peripheral edema, hypogonadism, and skin changes. In this setting, the early diagnosis and effective management of POEMS syndrome will prevent the burdensome disability of this disease. 
